# Purkinje cell models: past, present and future

**DOI:** 10.3389/fncom.2024.1426653

**Published:** 2024-07-10

**Authors:** Elías Mateo Fernández Santoro, Arun Karim, Pascal Warnaar, Chris I. De Zeeuw, Aleksandra Badura, Mario Negrello

**Affiliations:** ^1^Department of Neuroscience, Erasmus MC, Rotterdam, Netherlands; ^2^Netherlands Institute for Neuroscience, Royal Academy of Arts and Sciences, Amsterdam, Netherlands

**Keywords:** Purkinje cell, neuron model, climbing fibers, cerebellum, synaptic plasticity, neuraldynamics, computational models, ion channels

## Abstract

The investigation of the dynamics of Purkinje cell (PC) activity is crucial to unravel the role of the cerebellum in motor control, learning and cognitive processes. Within the cerebellar cortex (CC), these neurons receive all the incoming sensory and motor information, transform it and generate the entire cerebellar output. The relatively homogenous and repetitive structure of the CC, common to all vertebrate species, suggests a single computation mechanism shared across all PCs. While PC models have been developed since the 70′s, a comprehensive review of contemporary models is currently lacking. Here, we provide an overview of PC models, ranging from the ones focused on single cell intracellular PC dynamics, through complex models which include synaptic and extrasynaptic inputs. We review how PC models can reproduce physiological activity of the neuron, including firing patterns, current and multistable dynamics, plateau potentials, calcium signaling, intrinsic and synaptic plasticity and input/output computations. We consider models focusing both on somatic and on dendritic computations. Our review provides a critical performance analysis of PC models with respect to known physiological data. We expect our synthesis to be useful in guiding future development of computational models that capture real-life PC dynamics in the context of cerebellar computations.

## 1 Introduction

**Figure 1:**
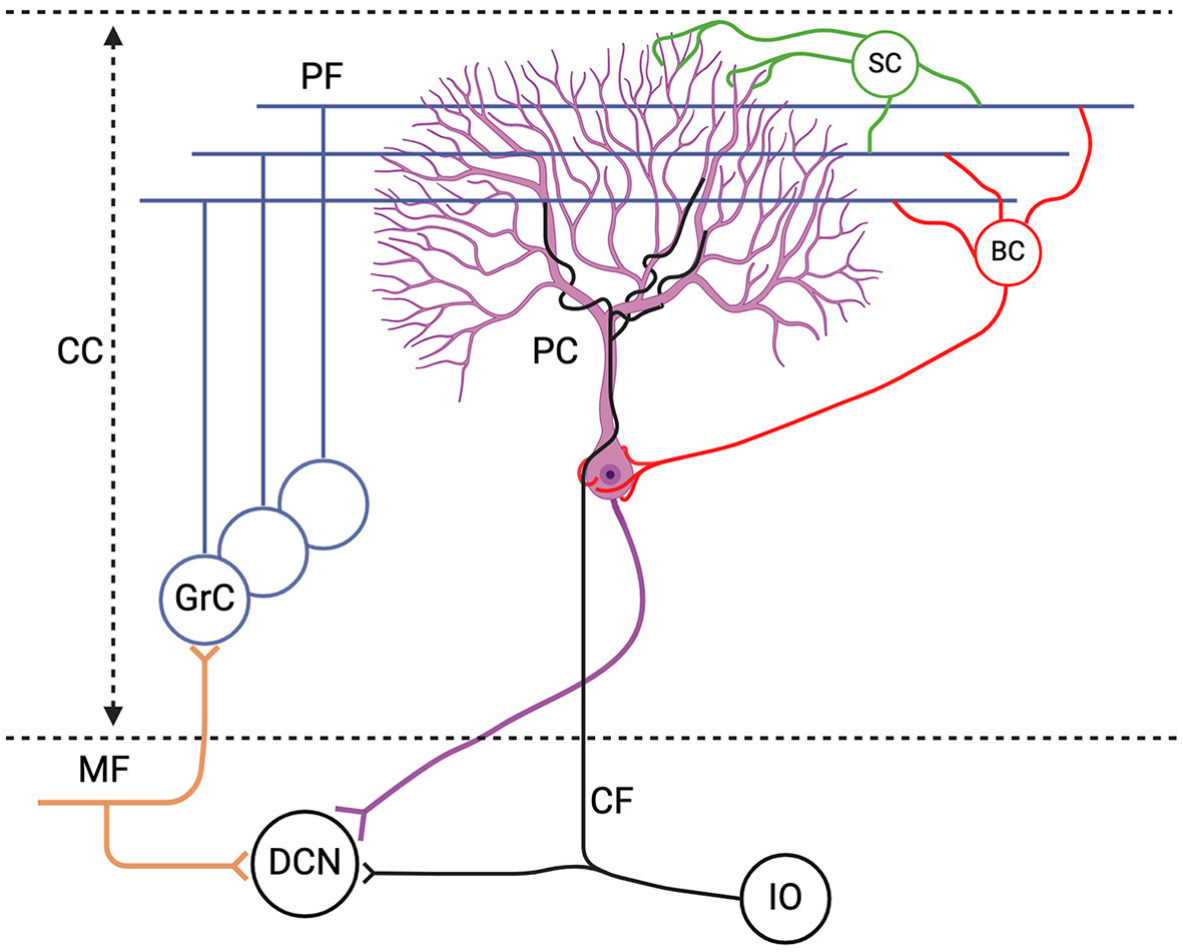
Schematic representation of the excitatory inputs to the Purkinje cell (PC). The PC receives excitatory inputs from: (1) parallel fibers (PF), axonal extension of granule cells (GrC) which receive excitatory signals from the mossy fibers (MF), and (2) climbing fibers (CF) from the inferior olive (IO); as well as inhibitory inputs from stellate cells (SC) and basket cells (BC) which are collectively referred to as molecular layer interneurons (MLIs). The only output of the cerebellar cortex (CC) is the PC axon which converges onto the deep cerebellar nuclei (DCN). Figure generated with BioRender.

The cerebellum plays a central role in motor learning and cognitive processes as it crucially contributes to motor coordination, precision and timing. Purkinje cells (PC) receive all incoming sensory and motor information and generate the sole output of the cerebellar cortex (CC) (Figure 1). PCs serve as pivotal mediators, receiving and processing diverse and rich sensory and motor information. For example, it has been shown that during the eyeblink conditioning task—a classical associative learning paradigm where a previously inert stimulus, such as light, is paired with a salient air puff to evoke eyelid closure—PCs adaptively adjust their firing rates based on the timing of the stimulus by integrating input from the inferior olive (IO), eliciting blinks (Gilbert and Thach, 1977; ten Brinke et al., 2015; Cook et al., 2021). In fact, there are several examples of sensorimotor behavior where a timely adaptation of PC activity is crucial for the correct execution of movement (Gao et al., 2012). There is consensus in the field of neuroscience that the investigation of PC's responses and plasticity is essential for understanding the cerebellar circuit and motor learning.

The high degree of morphological homogeneity of the CC across vertebrate species hints at a shared computational framework. Physiologically, PCs seem to produce a wide spectrum of spiking activity involving tonic firing, different types of bursts and wide distribution of pauses. Part of this spectrum can be related to differences in excitability in connection with Aldolase C, a glycolytic enzyme related to energetic metabolism of the cell (Voogd and Ruigrok, 2004; Zhou et al., 2014). The fact that cells can be categorized in two classes—Zebrin positive (Z+) and Zebrin negative (Z-)—invites a deeper exploration of the nuanced intricacies of PC dynamics, via computational models. Notably, experimental studies have pointed to other regional differences in the cerebellum such as: differences in the connectivity patterns between PCs and GrCs across anterior and posterior lobules (Guo et al., 2016) and different PC morphologies (Busch and Hansel, 2023). For all of these divergent properties, the biophysical models can help identify the crucial parameters that distinguish these PC populations, and potentially relate them to the morphological and molecular differences.

In the mammalian nervous system, the PC stands out among the most complex and largest of neurons (Sugimori and Llinás, 1990; Miyakawa et al., 1992). Discovered in the 19th century by Jan Evangelista Purkinje (Zárský, 2012), its unique structure was later described by Golgi and Cajal. The dendritic arbor of the PC is highly elaborate, consisting of numerous branching dendrites that have a surface area 100x the size of the cell body. The PC membrane has a complex set of ion channels and gating mechanisms that regulate its activity (Raman and Bean, 1999). It emits two distinct kinds of action potentials (AP): simple spikes (SS), triggered by mossy fiber signals (MF) which are relayed via granule cells (GrC) and reach PCs via the parallel fibers (PFs), and complex spikes (CS) which are triggered by the CF input from the IO (Schmolesky et al., 2002) (Figure 1). The SS activity is also modulated by the inhibitory MLI input (Wulff et al., 2009). Experimental studies have demonstrated substantial voltage-dependent dendritic conductances in the PC, emphasizing the independent contribution of the soma and the dendritic arbors to PC activity (Llinás and Sugimori, 1980b; Davie et al., 2008).

These unique features have drawn significant attention to the PC, establishing it as one of the most frequently modeled neurons in the brain. Over the years multiple PC models have been developed, ranging from highly detailed morphologies with hundreds of compartments to more simplified models. Understanding the intricate dynamics of PC—spiking patterns, trimodal firing patterns, current x frequency behavior, multistable dynamics, plateau potentials, plasticity dynamics, and input/output computations—is crucial for unraveling the complexities of cerebellar function and advancing our knowledge of motor learning and cognitive processes. It is imperative to conduct a thorough examination of modern models to foster the future development of computational PC models. This review aims to fill this need by providing an overview of contemporary PC models. Our objective is to facilitate the critical analysis of existing proposals and ideas, enabling researchers to adapt and redesign models based on current neurophysiological and molecular data. The ultimate goal is to offer a comprehensive understanding of the relationship between PCs' dynamics and functions, allowing for the contextualization of future models and providing an overview of the current state of thought in the field.

## 2 Purkinje cell models

Several types of PC models exist, which we will group into a few categories. First, we distinguish between detailed (>10 compartments) and simplified (< 10 compartments) models (Tables 1, 2). As is the case for any type of model, the choice of level of detail depends on the research question(s) being tackled. Detailed models attempt to capture the rich phenomenology of PCs, while simplified models of reduced complexity capture either a specific part of behavior or explain a given phenomenon in abstract terms, often resorting to dynamical systems theory. At least two more levels of analysis are crucial to understand the role of the CC in motor control, the plasticity of the PC in response to the GrC input and the activity of PCs across cerebellar networks at large. These levels give rise to the categories of synaptic and network models (Tables 3, 4). Importantly, experimental data has shown that connections with molecular layer interneurons (MLIs) are also plastic (Bao et al., 2010; Brown et al., 2019; Arlt and Häusser, 2020), but these properties have not yet been included in most of the PC models. Finally, we give an overview of a category of more traditional, abstract models based on the perceptron analogy, or what is now called a feed forward neural network. Perceptron-like models are useful to quantify metrics like the information capacity (in bits) of the PC to encode patterns (Table 5).

**Table 1:** Summary of selected detailed models.

**References**	**Dynamics**	**Main characteristics**		
		**Morphology**	**Experimental data**	**Description**
Pellionisz and Llinás (1977)	HH type	Frog 62 compartments	Na^+^ and K^+^ conductances from Hodgkin and Huxley (1952) HH parameters based on data from Llinas et al. (1969) and Hardy (1973)	Demonstrates simple passive dendritic integration
Shelton (1985)	HH type	Guinea-pig and rat 1089 compartments	Parameters based on *in vitro* intracellular recordings from Pellionisz and Llinás (1977), Scholfield (1978), Glantz and Viancour (1983), and Lev-Tov et al. (1983)	Calculated membrane resistivity in a passive multi-compartmental model
Bush and Sejnowski (1991)	Markov type	Rat from (Shelton, 1985) 1089 compartments	Conductance for Na^+^ channels based on patch clamp data from mammalian PCs [neuroblastoma cell line, rat myotubes and a pituitary cell line (Aldrich et al., 1983)] Passive membrane parameters from Shelton (1985)	Voltage-dependent conductances, significantly simpler than HH kinetics
De Schutter and Bower (1994a)	HH type Ca^2+^ dynamics	Guinea-pig from (Rapp et al., 1992, 1994) 1600 compartments	Passive membrane parameters from Rapp et al. (1992) Voltage-dependent ionic conductances based on: *in vitro* studies (Llinás and Sugimori, 1980a,b; Hounsgaard and Midtgaard, 1988); voltage-clamp studies (Vandenberg, 1987; Kaneda et al., 1990; Regan, 1991; Wang et al., 1991), and single-channel studies (Gähwiler and Llano, 1989; Gruol et al., 1991) Channel kinetics based on (Connor and Stevens, 1971; Llinás and Sugimori, 1980a; De Schutter, 1986; Spain et al., 1987; Hirano and Hagiwara, 1989; Yamada et al., 1989; Kaneda et al., 1990; Regan, 1991)	Ten different ion channels differentially placed in soma, smooth and spiny dendrites Somatic spiking relation to current injection (no spontaneous spiking) Different role for the arbor and the soma
Miyasho et al. (2001)	HH type Ca^2+^ dynamics	Guinea-pig from (Shelton, 1985) 1088 compartments	Based on data presented in DB model (De Schutter and Bower, 1994b)	Modified DB model Added low threshold channel types Sensitivity to Ca^2+^ lowered Replicates *in vitro* behavior of Ca^2+^ spikes in dendrites
Forrest (2014)	HH type Ca^2+^ dynamics TTX-sensitive current using Ohm's law	Rat from Shelton (1985) Guinea-pig from Rapp et al. (1994) 1089/41/5 compartments	Somatic PC channel dynamics obtained from voltage-clamp current measurements from Khaliq et al. (2003) Dendritic PC channel dynamics obtained from Miyasho et al. (2001)	Soma model from Khaliq et al. (2003), added SK Ca^2+^ channel Dendritic model from Miyasho et al. (2001), added Ih and Ihvk channels Na/Ca^2+^ exchanger with extracellular K concentration and intracellular Ca^2+^ concentration Shows trimodal firing pattern when input is compromised Replicates results when blocking Kv1.2 channels Reduction algorithm from Bush and Sejnowski (1991)
Anwar et al. (2013)	Stochastic ion channels	100 compartments	P-type Ca^2+^ from Usowicz et al. (1992), Swensen and Bean (2005), and Anwar et al. (2012) Other parameters from Bhalla and Iyengar (1999), Kuroda et al. (2001), and Tanaka et al. (2007)	Dendritic compartment model with optimized Ca^2+^ diffusion Stochastic intracellular Ca^2+^
Forrest (2014)	HH type Ca^2+^ dynamics	Rat from Shelton (1985) 41 compartments	Simultaneous somatic and dendritic whole-cell patch-clamp data from Miyasho et al. (2001), Khaliq et al. (2003), and Forrest et al. (2012)	Reduced model from Forrest et al. (2012)
Masoli et al. (2015)	HH type Ca^2+^ dynamics	Guinea-pig from Rapp et al. (1992, 1994) 1,600 compartments (1 somatic and 1,599 dendritic)	Passive properties from De Schutter and Bower (1994b); Na^+^ channels dynamics based on Raman and Bean (2001); K^+^ channels from Courtemanche et al. (1998), Raman and Bean (2001), Khaliq et al. (2003), Akemann and Knöpfel (2006), Diwakar et al. (2009); Calcium dependent K^+^ channels (Rubin and Cleland, 2006; Solinas et al., 2007; Anwar et al., 2012); Ca^2+^ channels (Huguenard and McCormick, 1992; Swensen and Bean, 2005; Xu and Clancy, 2008; Anwar et al., 2012); H channel (Angelo et al., 2007; Larkum et al., 2009); Ca^2+^ buffer (Anwar et al., 2012)	Modified DB model, Extensive channel dynamics from Khaliq et al. (2003), Akemann and Knöpfel (2006), and Anwar et al. (2012), among others. Current transfer between axon and dendrite allowed for spontaneous SS and CS
Zang et al. (2018)	HH type Ca^2+^ dynamics	Wistar rat from Roth and Häusser (2001) 4 compartments	Channel dynamics were based on many studies: Na^+^ (Khaliq et al., 2003); H (Angelo et al., 2007); T-type Ca^2+^ (Otsu et al., 2014); P-type Ca^2+^ (Benton and Raman, 2009); Ca^2+^ (Anwar et al., 2012, 2014; Indriati et al., 2013); BK (Anwar et al., 2012; Benton et al., 2013); SK2 (Hirschberg et al., 1998); Kv3 (Martina et al., 2007); Kv4 [experimental data of Dieudonné and (Otsu et al., 2014)]; Kv1 (Otsu et al., 2014)	Designed as a modern update to the original DB model. Calculable ATP cost of spiking due to energy input associated with dynamics

**Table 2:** Summary of selected simplified models.

**References**	**Dynamics**	**Main characteristics**	
		**Experimental Data**	**Description**
Raman and Bean (2001)	Markov scheme	Voltage-clamp recording of Na^+^ channels in mice (original data)	Resurgent Na^+^ dynamics model
Genet and Delord (2002)	HH type circuit model	PC dynamics based on Gähwiler and Llano (1989), Gruol et al. (1991), Yuen et al. (1995), and Jacquin and Gruol (1999)	Simplified dendrite model Replicates transition from Ca^2+^ plateaus to valleys
Khaliq et al. (2003)	HH type TTX-sensitive current using Ohm's law	Voltage-clamped current measurements from heterozygous Scn8a^med^ mice (original data)	21 ion channels, added tetrodotoxin-sensitive Na^+^ channel kinetics (mainly NaV1.6 channel that has two transition routes between inactivated and recovery of the channel) Simulates Ca^2+^ concentration in a 100 nm shell under the membrane
Loewenstein et al. (2005)	3 ionic currents dynamical systems model	Cell-attached and whole-cell patch-clamp from Sprague-Dawley rats and Dunkin-Hartley guinea pigs (original data)	An instantaneous, non-inactivating inward current (Na^+^ current) A slow h-like current and a voltage-independent outward current
Akemann and Knöpfel (2006)	Molecularly defined channel entities	Single-unit recordings, whole-cell patch- and dynamic-clamp recording from Kv3.3 KO mice and wild types raised on a mixed 129/Sv, C57BL, and ICR genetic background (original data)	Resurgent and non-resurgent Na^+^ channels Molecular defined K^+^ hyperpolarization current
Fernandez et al. (2007)	HH type Channel dynamical system model	Whole-cell current- and voltage-clamp recordings from Sprague-Dawley rats (original data)	Bistable model of 5 equations (3 channels) that can reproduce tonic firing Simplified model with 2 channels
Genet et al. (2010)	Complex nonlinear membrane PDEs	Current- and voltage-clamp recordings from mice, rats and guinea pigs (Shelton, 1985; Barbour, 1993; Vincent and Marty, 1996; Etzion and Grossman, 1998, 2001; Xia et al., 1998; Jaeger and Bower, 1999; Bushell et al., 2002; Womack and Khodakhah, 2002a; Angelo et al., 2007)	Full morphology model based on the GD model Simplified dendrite model (Raman and Bean, 2001; Khaliq et al., 2003)
Anwar et al. (2012)	HH type	PC channel dynamics obtained with patch-clamp experiments from Cox et al. (1997), Hirschberg et al. (1998), Maeda et al. (1999), Swensen and Bean (2005), and Iftinca et al. (2006)	Dendritic single-compartmental model with Diffusion compensating mechanism to replace Ca^2+^ radial diffusion Ca^2+^ channels of P- and T-type and Ca^2+^-activated K^+^ channels of BK- and SK-type
Buchin et al. (2016)	IF type	Whole-cell current-clamp and single patch-clamp recordings from Sprague-Dawley rats (original data)	Bistable adaptive exponential integrate-and-fire model (aEIF) SS can be inhibited through inverse stochastic resonance
Burroughs et al. (2020)	HH type Markov schemes (for channel dynamics)	Na^+^ channels from Raman and Bean (1997, 2001), Khaliq et al. (2003), Khaliq and Raman (2006) K^+^ channels based on Stuart and Häusser (1994) CS rate from Davie et al. (2008)	2 models: 5/3 ion channels CF input simulated as 2 decaying exponentials as found in Llinás and Sugimori (1980b) and Davie et al. (2008)

**Table 3:** Summary of selected synaptic models.

**References**	**Dynamics**	**Main characteristics**	
		**Experimental data**	**Description**
Fiala et al. (1996)	Mathematical model	mGluR1, G-protein, PLC, IP_3_, DAG and PIP_2_ from Sprague-Dawley rats (Blackstone et al., 1989) Ca^2+^-dependent K^+^ conductance from rats (Khodakhah and Ogden, 1993) Membrane and reversal potentials from De Schutter and Bower (1994a)	Dynamics of glutamate-activated mGluRs activation Eye-blink learning shown only in PCs where the Ca^2+^ response coincides with the production of cGMP in the cytoplasm, triggered by US evoked CF input
Doi et al. (2005)	Kinetic Ca^2+^ dynamics	Parameters from *in vivo* experiments from Bezprozvanny et al. (1991), Khodakhah and Ogden (1995), Marchant and Taylor (1997) and Fujiwara et al. (2001)	Dendritic spine model with 3 compartments: cytosol, postsynaptic density (PSD), and endoplasmic reticulum (ER) Showed regenerative Ca^2+^ release via IP_3_ receptors (IP_3_r)
Steuber et al. (2006)	Mathematical model 2 ODEs	From Fiala et al. (1996)	Simplified from Fiala et al. (1996) Glutamate increase triggers Ca^2+^
Tanaka et al. (2007)	IF type Ca^2+^ dynamics	Whole-cell patch-clamp recordings rats or mice (original data)	Leaky integrate-and- fire (LIF) model LTD of single synapse based on (Bhalla and Iyengar, 1999)
Brown et al. (2011)	HH type Biomechanical	Leak current from Rapp et al. (1994) Ion channel kinetics from Miyasho et al. (2001)	Combination of Brown et al. (2008), Bhalla and Iyengar (1999), and Miyasho et al. (2001) Second model has a reduced arbor for computational efficiency Biochemical PIP_2_ and IP_3_ pathways lead to LTD
Antunes and De Schutter, 2012	Stochastic model Ca^2+^ dynamics	Experimental results from Schmidt et al. (2003), Doi et al. (2005), Kyriakis (2007), Tanaka et al. (2007)	Stochastic signaling network with positive Ca^2+^ feedback loop Positive feedback loop with AMPA receptors Different LTD for different parameters
Anwar et al. (2014)	Ca^2+^ dynamics	Ca^2+^ dynamic parameters from Anwar et al. (2012)	Dendritic simulation model analysis (different diameters of dendritic models) Intracellular Ca^2+^ diffusion
Hepburn et al. (2017)	Stochastic model Ca^2+^ dynamics	PC whole-cell patch-clamp recordings from mice (original data)	Cerebellar model based on Antunes and De Schutter (2012) and Bhalla and Iyengar (1999) Updated Ca^2+^ dynamics that are closer to experimental data Additional dynamics for rapidly accelerated fibrosarcoma (Raf) kinase and Raf kinase inhibitor protein (RIPK)
Narain et al. (2018)	Probabilistic model	GrC data from Giovannucci et al. (2017) and Wagner et al. (2017) Eyeblink conditioning data (Siegel et al., 2012; ten Brinke et al., 2015)	Used a type of learning which approaches a Bayesian least squares estimator LTD when GrCs and CFs are active and LTP when GrCs are active and no CF activity
Zamora Chimal and De Schutter (2018)	Stochastic model Ca^2+^ dynamics	Parameters from Hepburn et al. (2017) CaMKII characteristics from Kubota and Bower (2001)	Extension of Hepburn et al. (2017) and Bhalla and Iyengar (1999) with Ca^2+^/calmodulin-dependent protein kinase II (αCaMKII) dynamics Mechanistic CaMKII activation and phosphorylation. Stochastic PC model (bistable)
Majoral et al. (2020)	Molecular interactions Protein dynamics Ca^2+^ dynamics	Ca^2+^ oscillations from Somogyi and Stucki (1991) PKA pathway dynamics from Violin et al. (2008)	Ca^2+^ oscillations combined with concentration equations of mGluR7, G-protein coupled receptors (GPCRs), G protein signaling (RGS), G alpha stimulatory protein (Gs), inhibitory G protein (Gi), protein kinase A (PKA), adenylyl cyclase (AC) Molecular interactions: Ca^2+^, IP_3_ and IP_3_r
Mandwal et al. (2021)	HH type Biomechanical equations	All values except GIRK from Fernandez et al. (2007) GIRK from Bichet et al. (2003)	Biochemical mechanistic model based on Fernandez et al. (2007) and Bhalla and Iyengar (1999) Inward rectifier K^+^ (GIRK) ion channel added Time interval learning based on biomechanical principles of G-protein receptors

**Table 4:** Summary of selected network models.

**Reference**	**Dynamics**	**Main characteristics**		
		**Network**	**Experimental data**	**Description**
Jaeger (2003)	HH type Ca^2+^ dynamics	PC and ~175,000 PFs	PC properties from De Schutter and Bower (1994b)	PC DB model
Santamaria and Bower (2005)	HH type Ca^2+^ dynamics	PC, 1600 GrCs and PFs and MLIs	PC properties from De Schutter and Bower (1994b)	PC DB model MLIs are only SC
Maex and Steuber, 2013	HH type Ca^2+^ dynamics	50 PCs, 500 MLIs and 7100 PFs	PC Properties from De Schutter and Bower (1994b); Synaptic parameters from Solinas et al. (2006)	PC DB with axon for spike threshold attenuation (Maex and De Schutter, 2007) Adapted channel densities give model spontaneous activity
Clopath and Brunel (2013)	Rates Waveforms	PCs, MFs, GrCs, MLIs and MVN cells	*In vivo* recordings from VOR experiments in C57BL/6 mice and PC-Δγ2, GC-ΔKCC2 and PC-ΔKCC2 mice (Badura et al., 2016) (original data)	Plasticity at PF-PC and MF-MVN
Grangeray-Vilmint et al. (2018)	IF type	PCs, GrCs and MLIs	Heterozygous Thy1-ChR2-eYFP mice PC whole-cell voltage-clamp (original data)	PC (LIF model) PC with synapse transient STD mechanisms from Tsodyks and Markram (1997) and Bhalla and Iyengar (1999) STD alters how the PC responds to GrC input, from it being inhibitory to excitatory Allows for the same input to differentially affect groups of PCs
Luque et al. (2019)	HH type Ca^2+^ dynamics IF type	20 PCs, 2 IOs (CFs), 100 MFs, 2,000 GrCs, and 2 MVN cells	PC parameters from field potential recordings on C57B/6 mice (Middleton et al., 2008)	Reduced PC model based on Miyasho et al. (2001) and all other neurons are LIFs LTD/LTP at PF-PC, MF-MVN and PC-MVN
Tang et al. (2021)	IF type	50 PCs, 1,000 GrCs, 500 MLIs and 500 MFs	All cell parameters from whole-cell patch-clamp experiments (de Solages et al., 2008; Jelitai et al., 2016; Zampini et al., 2016; Grangeray-Vilmint et al., 2018)	Each PC receives from 100 GrCs and 8 MLIs and each MLI receives from 4 GrCs
Luque et al. (2022)	HH type Ca^2+^ dynamics IF type	200 PCs, 2,000 GrCs, 100 MFs, 200 IO/CF and 200 MVN cells	Parameters from Luque et al. (2019)	PC from Miyasho et al. (2001) and Luque et al. (2019) All other neurons are LIFs
Binda et al. (2023)	IF type	PCs, GrCs and MLIs	Whole-cell patch-clamp and loose cell-attached recordings Photostimulation (original data)	Network from Grangeray-Vilmint et al. (2018)
Geminiani et al. (2024)	IF type	PCs, GrCs, PFs, Golgi cells (GCs), BCs, SCs, MF, DCN and IO cells	Based on eyeblink conditioning data from ten Brinke et al. (2015), Ten Brinke et al. (2017), and Boele et al. (2018)	All neurons are extended-generalized LIFs (EGLIFs) Downbound (Z- PCs) and upbound (Z+ PCs) zones Plasticity at PF-PC and PF-MLI

**Table 5:** Summary of selected Perceptron-like models.

**Model**	**Dynamics**	**Main characteristics**	
		**Experimental data**	**Description**
Steuber and Schutter (2001)	HH type	PC properties from De Schutter and Bower (1994b)	DB PC model and artificial neural network (ANN) model ANN outperformed DB by an order of magnitude
Brunel et al. (2004)	Perceptron type	—	Abstract single layer learning perceptron model Feedforward neural network with excitatory synapses (50% silent synapses)
Steuber et al. (2007)	HH type	PC properties from De Schutter and Bower (1994b)	Modified DB model taking ANN input for synaptic weights. Learns hyperpolarizing Ca2+ causes pause
Walter and Khodakhah (2009)	Perceptron type	PC whole-cell recording and GrC electrical stimulation from Wistar rats (original data)	Artificial neural network based on experimental data
Clopath et al. (2012)	Perceptron type	—	Abstract single layer learning perceptron model Binary feedforward neural network model (bistability occurs)
Clopath and Brunel (2013)	Perceptron type	—	Feedforward neural network reaches maximal decoding capacity at 50%+ silent synapses Allows for more physiological output patterns (frequency trains)

### 2.1 Detailed models

In the 1970s, the increasing understanding of the composition, dynamics and cellular structure of the PC led to models incorporating high levels of biological detail. This development was largely possible thanks to the detailed Purkinje cell morphologies revealed by Golgi (Pellionisz and Llinás, 1977; Shelton, 1985) and horseradish peroxidase stainings (Rapp et al., 1994) as well as the advancements in the electrophysiological recordings. The integration of information in the complex dendritic tree of the PC was a focus of the early models, which incorporated passive electrical properties and detailed information about active ion channels kinetics. Some of the earlier models studying these properties with a realistic full scale morphology were based on the frog PC anatomy (Pellionisz and Llinás, 1977), or that of a guinea pig PC (Shelton, 1985). The Shelton model used Hodgkin-Huxley (HH) equations for channel kinetics, which at that time were gaining popularity among their contemporaries but still lacked experimentally verified parameters and were very computationally demanding. Bush and Sejnowski (1991) introduced Markov schemes to model channel kinetics, while using the same morphology as Shelton. Following these two, a landmark PC model was developed by De Schutter and Bower—the DB model (De Schutter and Bower, 1994a,b) comprising 1,600 compartments—this large detailed model was reconstructed from earlier morphological observations (Rall, 1964). Ten different ion channels were differentially distributed over the soma, the smooth dendrites and the spiny dendrites. Channel kinetics were computed using HH equations, using parameters observed in different *in vitro* voltage-clamp studies done in different animal species and different neuronal cell types. One of the model's breakthrough accomplishments was an accurate reproduction of spiking frequency to current injection amplitude. The model started to fire abruptly with a minimum firing frequency around 30–40 Hz, after which it produced linear firing rate increases up to saturation. This very closely reproduced data recorded *in vitro* from acute cerebellar slices (Raman and Bean, 1999). Dendritic and somatic spiking features were effectively captured by the model explaining the dynamics of increase of Ca^2+^ concentration over the dendritic arbor upon CF input, which had previously been shown experimentally (Miyakawa et al., 1992).

Miyasho took the DB model as their starting point and implemented new experimental insights into PC physiology, resulting in a 1,088 compartmental model (Miyasho et al., 2001). Several ion channel kinematic equations were modified, such as the P-type Ca^2+^ channels and delayed rectifier potassium (K^+^) channels. Furthermore, two low threshold channels, class-E Ca^2+^ and D-type K^+^ channels, were added to the model. Sensitivity of Ca^2+^ activated K^+^ channels was lowered compared to the DB model, allowing repetitive Ca^2+^ spiking. These updates made the PC replicate *in vitro* behavior, where, upon blocking voltage dependent sodium (Na^+^) channels with tetrodotoxin (TTX), the cell showed repetitive Ca^2+^ firing (Miyasho et al., 2001). The Miyasho model especially reliably mimicked the dynamics around the generation of Ca^2+^ spikes in the dendrites, replicating pharmacologically induced changes of channel dynamics seen *in vitro* (Llinás and Sugimori, 1980b). However, despite the updates, clear differences still remained with respect to experimentally measured PC physiology. Specifically, (1) the intracellular Ca^2+^ concentrations were not realistically estimated, and (2) the sensitivity to calcium of Ca^2+^ activated K^+^ channels was incorrectly estimated, and the channel distributions were considered uniform over dendrites. Furthermore, the complexities of axial and radial calcium diffusion were not incorporated in this model. Those were later shown to account for CS waveform variability, and non-linear dendritic spiking and signal amplification in another detailed PC model published in 2013 (Anwar et al., 2012).

The dendritic model of Miyasho was updated in 2012 to simulate 21 gated ion channels using the morphology earlier published by Shelton (Forrest et al., 2012). Forrest combined two models to make a 1,089 compartmental model: Miyasho's dendritic channels with a highly detailed dendritic model, and the somatic model from Khaliq (Khaliq et al., 2003). The latter simulated TEA-sensitive, TEA-moderately sensitive and TEA-insensitive voltage-gated K^+^ currents, BK voltage-gated Ca^2+^-gated K^+^ channels, hyperpolarization-activated mixed cation current (Ih), I-leak, resurgent Na^+^ current, P-type Ca^2+^ currents as well as intracellular Ca^2+^ dynamics. Moreover, the authors added the SK Ca^2+^-gated K channels to the soma, previously modeled in the midbrain neurons (Komendantov et al., 2004). Forrest also incorporated the Ih currents (Saraga et al., 2003), as well as the IKv1 channels (Akemann and Knöpfel, 2006), to the dendritic channels of the Miyasho model. Further additions by Forrest were: the inclusion of the Na^+^/K^+^ pumps (both Na^+^ and voltage dependent), with slight dynamical differences between the pumps located in the soma and the dendrites; a Na^+^/Ca^2+^ exchanger; an abstract representation of intracellular Na^+^ and extracellular K^+^ concentrations. Notably, Forrest's model was constructed to explain activity of isolated PC's from *in vitro* studies, in which synaptic inputs were compromised. PCs in this disembodied state show a repeating trimodal firing pattern, alternating between tonic firing, bursty firing and quiescence (Womack and Khodakhah, 2002b, 2003, 2004). Furthermore, this model incorporated Na^+^ spikes in the soma and Ca^2+^ spikes in dendrites and replicated experimental results in which pharmacological block of Kv1.2 channels increased the Ca^2+^ spike frequency in the dendrites and lowered the number of somatic spike bursts in the trimodal pattern. Forrest's results uncovered the intracellular PC dynamics governing the trimodal spiking phases, showing the impact of the Na^+^/K^+^ pump in trimodal firing and replicated *in vitro* results in which the pump was pharmacologically blocked. In the same paper, Forrest also reduced the 1089 compartmental model to two simpler models-−41 and 5 compartments respectively—being able to show the same spontaneous firing in the trimodal pattern activity. Forrest further expanded the 41 compartmental model in 2014, where he described a PC model with modifications to the implementation of the tonic to burst transition in the trimodal pattern by removal of one Na^+^/K^+^ pump and of the SK channel, as well as adding other HH dynamics (Forrest, 2014). This model reproduced the quiescent periods as observed *in vivo* (Loewenstein et al., 2005), and showed that CF input could dictate both the timing and duration of these periods.

Another model based on a detailed reconstruction of the PC morphology was proposed in 2015 (Masoli et al., 2015). It presented an extensive multicompartmental PC model with an intricate axonal compartment. The authors added Axonal Initial Segments (AIS), the site of action potential (AP) initiation located at the base of axons (Somogyi and Hámori, 1976). Moreover, the Masoli model also included three nodes of Ranvier (RN) separated by myelinated sections. This detailed axon representation was modeled with a set of 15 different subtypes of Na^+^, Ca^2+^ and K^+^ ionic channels, combined with internal Ca^2+^ dynamics. The authors showed that the firing of the PC is intrinsic, and relies on Na^+^ channel dynamics in the AIS, and P-type Ca^2+^ channels in the dendrites. Absence of either one of these channel types caused the model to become bistable, resulting in the possibility of the PC to switch to a quiescent state, as had been observed in anesthetized mice (Loewenstein et al., 2005). Masoli's model also exhibited a switch from tonic firing to complex bursting at higher input frequencies in response to high current injections, corroborating experimental findings (Kim et al., 2012). Intriguingly, in Masoli's model the APs could be triggered during complex bursts by dendritic depolarization currents. This could imply secondary spike generation in RNs, though it currently lacks experimental verification.

In 2018, Zang et al. (2018) published a substantially modified model based on the DB model. Here, the authors extensively updated the model and tuned it to recent experimental data, integrating new knowledge about ionic channels distributions and Ca^2+^ diffusion. Among other predictions, this allowed the authors to calculate the metabolic cost of PC activity. In the Zang model, CS consumed more adenosine triphosphate (ATP), resulting in the energy equivalent of ~40 SS. This high energetic cost of CS activity might help explain the negative correlation found experimentally between the firing rates of SS and those of CS (Cerminara and Rawson, 2004). This fact could reflect a homeostatic, protective mechanism inside the cerebellum to abide by its limited energy supply. The model also showcased that voltage-dependent K^+^ currents in the PC dendrites were essential in gating the spatial range of dendritic responses evoked by CFs. According to the Zang model, all synaptic inputs to the PC and the SS firing rate contribute to learning occurring inside the PC. Furthermore, the time interval between SSs and the CF input modulated the amplitudes of the spikelets after CF input, indicating that the response to CF input depends on the SS activity. According to this model, the dendritic arbors exhibit heterogeneous excitability, implying the existence of computational units at the level of individual branches. The question of PC dendritic compartmentalization was recently examined in more detail by the same authors (Zang and De Schutter, 2021). With slight changes to spiny dendrite conductances, this updated model was able to reproduce PF-evoked dendritic spike pauses similar to the experimental data (Rancz and Häusser, 2010), without affecting the findings of the 2018 version of the model. Individual dendritic branches could either exhibit linear or burst-pause coding behavior, showcasing the computational unit of the individual branch. According to the updated model, the PF-evoked dendritic spikes trigger Ca^2+^ influx in the branch, leading to long and/or short term depression (STD) at the PF-PC synapse. The modeled dendritic dynamics were context dependent, which allowed the PC to encode a wide range of behavioral properties by generating somatic SS burst-pause sequences when dendritic spikes occurred. When paired with CF input, Zang's 2021 model initiated cerebellar LTD in line with recent experimental findings (Rowan et al., 2018; Silva et al., 2024).

While detailed models are able to capture many aspects of PC dynamics, they are computationally expensive even today, a fact that limits their applicability to small- to medium-scale simulations and leads many researchers to focus on simplified modeling approaches.

### 2.2 Simplified models

The goal of the simplified models is to reduce complexity so as to capture either a specific part of PC spiking behavior or explain the phenomena in abstract terms. Usually this implies a heavily simplified morphology and/or ion channel make-up. This renders the models less biologically accurate but better suited to dynamical systems analysis of spike generation mechanisms, and in addition, due to reduced computational costs, usable in larger scale networks.

Khaliq, Gouwens and Raman constructed a single-compartment model of the soma to study the high-frequency SS firing observed in the PCs [(Khaliq et al., 2003); the KGR model]. This model is based on experimental results and previous modeling efforts from the same group that showed that resurgent Na^+^ currents were associated with a rapid recovery from inactivation and were thus important in sustaining high SS firing frequencies (Raman and Bean, 1997, 2001). The resurgent dynamics were incorporated in the model with TTX-sensitive Na^+^ channel kinetics, mainly the NaV1.6 channels. The authors decided to leave several ion channels out of their model. T-type and A-type currents were not incorporated due to the fact that their voltage-based activation and inactivation kinetics were unlikely to play a major role in spontaneous and current evoked firing of isolated PCs. This simplified model encompassed in total 8 ionic currents. The TTX-sensitive Na^+^ currents (of which the NaV1.6 channel was the main contributor) were calculated using Ohm's law, multiplying the ratio of open channels by the driving force and the maximal conductance observed (Raman and Bean, 1997). All other channels were modeled using HH simulations with parameters obtained from experimental data; the P-type Ca^2+^ current dynamics were modeled based on an older experimental dataset (Raman and Bean, 1999). The KGR model simulated the Ca^2+^ concentration in a 100 nm shell underneath the cell membrane. Resulting spontaneous SS firing was ~27 Hz, and the Na^+^ currents with a resurgent component contributed to PC excitability. Finally, the KGR model was used to simulate PC activity in an ataxic knockout mouse line lacking the expression of the NaV1.6 channels PCs. To simulate these mutant PC cells, the Na^+^ channels were tweaked to have faster inactivation rates and slower transition to a blocked state, so that they closely mimicked the recorded currents in mutant cells. This altered model indeed showed that a reduction in SS firing frequency depends on the Na^+^ current with a resurgent component, mediated by the NaV1.6 channels.

An adjusted version of the KGR model was later used to study the effect of a K^+^ channel, Kv3.3, on spontaneous firing in PCs (Akemann and Knöpfel, 2006). The authors updated the three voltage-gated K^+^ channels, used in the KGR model, to molecularly identified K^+^ channels. Furthermore, they included both the resurgent and non resurgent Na^+^ channels, which were simulated separately in the KGR model. The Akemann model also included a P-type Ca^2+^ current, a Ca^2+^-activated K^+^ current, and an Ih current, with conductances based on kinetics previously established by the KGR model. The K^+^ channels of the Kv1 and Kv4 type were simulated using HH equations. The Kv3.3 currents—characterized by high activation threshold, fast activation and deactivation—recapitulated findings from the dynamic patch clamp experiments. Among several important findings of this model, the simulations revealed that Kv3.3 currents could only substantiate the higher firing frequencies by cooperation with resurgent Na^+^ currents, inducing a tonic inward Na^+^ current which drove the intrinsic firing of PCs.

Simplified models were also developed to explore computational principles in the PC dendrites. Genet and Delord used a single-compartment model to simulate nonlinear dynamics underlying plateau potentials and Ca^2+^ spikes in the PCs dendrites [(Genet and Delord, 2002); GD model]. Plateaus are depolarizations that outlast the end of their triggering stimulus and last from tens of milliseconds to seconds (Llinás and Sugimori, 1980b). These plateaus, as well as dendritic spiking, rely on voltage dependent P/Q-Ca^2+^ channels. The GD compartmental model further included a simplified calculation of internal Ca^2+^ regulation and three ionic currents: P-type Ca^2+^ current, delayed-rectifier K^+^ current and a generic class of K^+^ channels activating sharply in the sub-threshold voltage range. It successfully replicated experimentally observed spontaneous transitions between Ca^2+^ plateaus and Ca^2+^ valleys, which for example the DB model failed to show (De Schutter and Bower, 1994a,b). Using a dynamical systems approach the GD model explained how the plateau and valley states in the dendrites were correlated to the input currents. Interestingly, the model's only Ca^2+^ channel was the P-type Ca^2+^ channel, in agreement with experimental data showing that indeed the P-type Ca^2+^ channels constitute the major part of the Ca^2+^ currents (Usowicz et al., 1992). In comparison, the detailed model of Miyasho discussed above, also included E and D type Ca^2+^ currents. The GD model showed that brief depolarizing currents could trigger long after-depolarizations that resemble experimental plateaus in the absence of the ‘long' inactivating time constants provided by the E and D type Ca^2+^ channels. Explorations of the model showed that slow intracellular Ca^2+^ increases in the sub-threshold voltage range decreased the Ca^2+^ Nernst potential, thereby reducing the magnitude of Ca^2+^ current on a time scale of hundreds of milliseconds. Notably the K^+^ channels in the GD model were generic, as the authors argued that the dendritic function of distinct K^+^ channel subclasses was not understood well enough to be modeled separately.

Interest in the Ca^2+^ channel dynamics in the dendritic arbor led to the development of a new single dendritic compartment model (Anwar et al., 2012), which was later computationally optimized to simulate realistic Ca^2+^ diffusion (Anwar et al., 2013). This updated model incorporated stochastic spatially arranged ion channels, and stochastic intracellular Ca^2+^ dynamics. Most full-scale models assume voltage-gated computations that represent average kinetics over many channels. For whole-cell models this is justified because fluctuations resulting from stochastic gating kinetics in small systems tend to become negligible. However, the stochastic behavior of channels in the PC dendrites, together with the complex morphology of the PC dendritic arbor led to high variability in dendritic spikes triggered by CF-mimicking input (Anwar et al., 2013). This high temporal and spatial variability in Ca^2+^ levels observed in dendritic arbor simulations could have great functional significance on e.g. short-term plasticity mechanisms.

As mentioned above in the description of the detailed models, the 2012 Forest model, also reduced the full 1089 compartment model to 2 simplified models of smaller morphological complexity to decrease the computational demand (Forrest et al., 2012). Both reduced models were able to conserve reproducibility of the full model with simpler channel compositions. The simplified models were used to study CF input-dependent changes in the stable-to-bistable PC activity state, as observed in experimental studies (Loewenstein et al., 2005). The reduced models explored the Ca^2+^ dynamics, in which intracellular Ca^2+^ concentration prior to the CF input determined the balance between excitation or hyperpolarization upon CF input. Forrest's simulations assumed a CF-specific activation of Ca^2+^ responsive SK K^+^ channels by means of channel co-localization, resulting in a hyperpolarizing current. If prior to the CF input, the neuron was tonically firing there was a high dendritic internal Ca^2+^ concentration. The CF input, opening Ca^2+^ channels, resulted in high Ca^2+^ concentrations leading to a large Ca^2+^ activated K^+^ current hyperpolarizing the PC, which then entered the quiescent state. In the quiescent state the Ca^2+^ concentration receded, and therefore the Ca^2+^ concentration in the dendrites upon CF input in this state is relatively smaller. This results in subsequent hyperpolarizing current through Ca^2+^ activated SK potassium channels being smaller than in the Akemann 2006 model (Akemann and Knöpfel, 2006).

More recently, two single-compartmental models were developed to examine if essential aspects of the PC CS-waveform can be generated by a limited number of somatic channels (Burroughs et al., 2020). The models examined were (1) a 5-current model which contained Ca^2+^, Na^+^ and K^+^dynamics; and (2) a reduced 3-current model, which only contained Na^+^ and K^+^ dynamics. The Na^+^ current was modeled after the Markovian scheme of Raman and Bean (1997) while the K^+^ current was modeled after the previously discussed Masoli model (Masoli et al., 2015). The Ca^2+^ dynamics in the 5-current model were based on the Miyasho model (Miyasho et al., 2001) with an addition of an SK channel implemented with dynamics from Gillies and Willshaw (2006). The final addition was a base input current combined with a CF input current. This CF current was modeled as a combination of two exponentially decaying inputs in line with experimental data on CF synaptic input (Davie et al., 2008). Both the 3- and 5-current models were able to simulate general CS behavior, causing a high amplitude of the initial spike followed by spikelets. Additionally, the 5-current model allowed for the CF input to influence the SS rate, causing depression after a CS and suggesting that the SK and Ca^2+^ dynamics are essential for CS induced depression as observed *in vivo* (Schmolesky et al., 2002).

#### 2.2.1 Bistability

*In vitro* experimental data show that the passive firing of PCs can follow a trimodal firing pattern of tonic firing, burst firing and quiescence (Llinás and Sugimori, 1980b; Raman and Bean, 1997). Bistable patterns toggling between tonic firing and quiescence have also been observed in anesthetized mice (Loewenstein et al., 2005) as well as awake cats (Yartsev et al., 2009). However, despite sparking interest guided by these initial observations, bistability—switching between prolonged depolarized (up) state, and periods of hyperpolarization (down state)—has never been reported as a correlate of behavior, nor has it been observed in awake behaving mice (Schonewille et al., 2006) as it seems to be modulated by anesthesia (Engbers et al., 2013). Nevertheless, *in vitro* experiments show that bistability is within the scope of PC dynamics, and gives clues to its dynamical responses.

A few reduced dynamical models have been used to explain bistability by using slow state variables such as Ih or Ca^2+^ currents (Loewenstein et al., 2005). Fernandez produced a model with only four voltage-gated conductances (Fernandez et al., 2007), and electrically coupled dendrites and soma, to reproduce the relative depolarized state of the dendrites during the AP measured in the soma (Stuart and Häusser, 1994; McKay and Turner, 2004). The Fernandez model depended on a saddle node bifurcation to account for transitions between rest and firing state (such as the low firing range < 40 Hz in the bistable range, expected from the dynamical system). This model displayed tonic activity with frequencies up to 150 Hz and replicated dynamical system characteristics underlying spike behavior as observed experimentally in PCs, such as a highly nonlinear I-V relationship near threshold and the generation of long delays to first spike compared to the average ISI time

To study the dynamical states of the PC, Fernandez and colleagues constructed a simplified two-channel model to investigate if the CF input could trigger state transitions in a bistable region (Engbers et al., 2013). This resulted in transitions from rest to firing, and vice-versa, together with a long delay for the first spike. The commanding factors underlying bistability in this model were: (1) the (large) membrane time constant, and (2) spike refractory dynamics that generated large enough depolarizing after potentials that triggered the following spikes leading to a persistent activity. The positive current flowing from dendrites into the soma was also found to contribute to bistability. The Ih current was not directly involved in establishing bistability dynamics, but rather limited the range of bistability in the phase plane. This somewhat contradicts model simulations by Loewenstein et al. (2005) that assumed I h to be a main player in establishing bistability.

In 2010 Genet expanded on the 2002 GD single-compartment simplified model adding a full dendritic morphology (Genet et al., 2010). Analysis performed with the 2010 Genet model suggests that the dendritic Ca^2+^ plateaus and valleys, triggered upon PF stimulation, respectively, causes periods of firing and silencing of PCs (bistable state).

An altered and reduced version of the original 2012 Forrest model also implemented bistable PC behavior (Forrest, 2014). Here, the authors altered the mechanisms of the tonic to burst transition using a D-Type K^+^ current, called I_D_ with an added inactivation rate modulation parameter (k), as well as additional mechanics for plasticity in the baseline intracellular Ca^2+^ concentrations. The updated Forrest model showed that PF inputs could alter the frequency of PC SS firing, but not the pattern of firing. Moreover, the updated internal Ca^2+^ dynamics allowed the simulated PCs to toggle between up and down states.

The most recent model of bistability was proposed by Buchin et al. using an experimentally tuned adaptive exponential integrate-and-fire model (aEIF) with type II excitability and nonlinear voltage dynamics (Buchin et al., 2016). In contrast to Forrest's models, Buchin's model relied upon an inverse stochastic resonance depending on noise variability for bistability rather than an internal Ca^2+^ dynamics. Whether these mechanisms best explain PC bistability remains an open question.

### 2.3 Synaptic models

**Figure 2:**
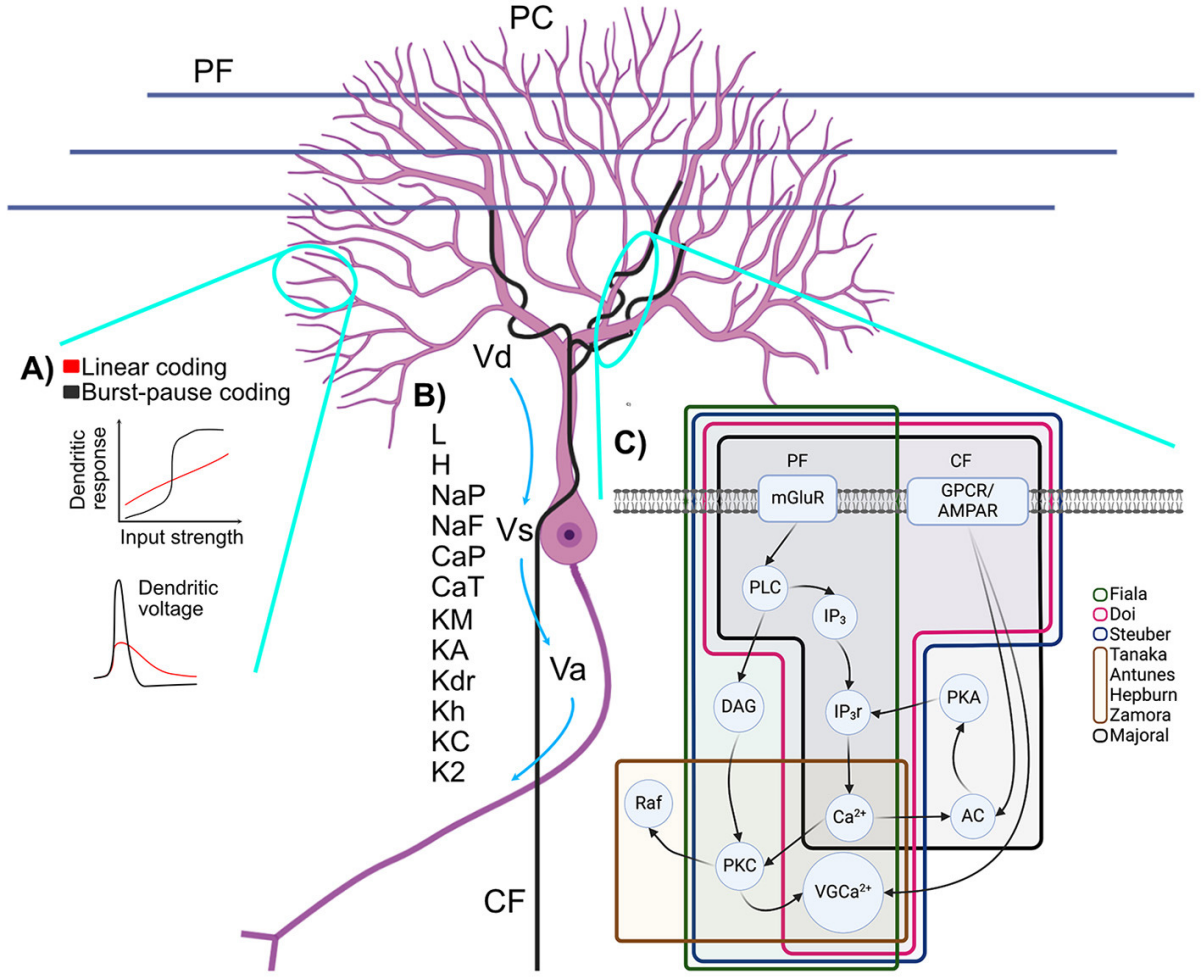
Schematic representation of **(A)** branch-dependent bimodal computations, **(B)** membrane potentials and ionic channels of PC models and **(C)** simplified molecular pathway involved in LTD and the PF-PC synapse. **(A) (TOP)** Multiple encoding mechanisms have been proposed for the PC: a linear firing rate response to increasing current and a pause duration mechanism. **(BOTTOM)** EPSCs in spiny dendrites are nonlinear and may or may not produce spikelets (adapted from Zang and De Schutter, 2021). **(B)** Dendritic (Vd), Somatic (Vs) and Axonic (Va) membrane potentials and their various ionic channels: leak current (L), hyperpolarization-activated current (H), persistent Na^+^ current (NaP), fast Na^+^ current (NaF), P Ca^2+^ current (CaP), T Ca^2+^ current (CaT), persistent K^+^ current (KM), A K^+^ current (KA), delayed K^+^ rectifier (Kdr), anomalous K^+^ rectifier (Kh), BK calcium-activated K^+^ current (KC), K2 calcium-activated K^+^ current (K2). **(C)** Starting from PF: mGluR activates the PLC enzyme which yields two products, IP_3_ and diacylglycerol (DAG). IP_3_ activates the opening of IP_3_r which liberates Ca^2+^. DAG and Ca^2+^ activate PKC. Ca^2+^ from CF and from IP_3_r stimulate AC which activates PKA, opening more IP_3_r through positive feedback. PKC and signals from GPCR or AMPAR from the CF activate voltage-gated Ca^2+^ channels (VGCa^2+^). Figure generated with BioRender.

Due to its imputed role in learning, plasticity at the PF synapses (Figure 2) has been the focus of multiple modeling forays. Whereas some models focused solely on the integration mechanisms in the PC dendritic arbor, others simulated the dynamics of the molecular pathways underlying the plasticity rules in dendritic spines. One of the first synaptic models examined how glutamate activates metabolic glutamate receptors (mGluRs), leading to cytoplasmic Ca^2+^ fluctuations, which vary greatly in timing across different PCs and potentially encode the timing for stimuli in classical eyeblink conditioning (Fiala et al., 1996). This model suggested that effective eyeblink learning occurred in PCs when Ca^2+^ response and cyclic guanosine monophosphate (cGMP) production were simultaneous, a result of cerebellar input. It also showed that the response delay—ranging from 100 ms to several seconds—is due to the sequential activation of phospholipase C (PLC), the generation of inositol (1,4,5)-trisphosphate (IP_3_), and the release of IP_3_-induced Ca^2+^. Steuber later simplified this model, identifying critical elements like Ca^2+^ dependent feedback and autocatalysis that influence these delays, which also vary with mGluR density (Steuber et al., 2006). In parallel, efforts were made to refine the dendritic model to explore the synaptic detection input coincidence and the role of the IP_3_-induced Ca^2+^ release in synaptic plasticity (Doi et al., 2005).

A highly detailed biochemical dynamical model of long-term depression (LTD) at the PF-PC synapse was introduced in 2012 (Antunes and De Schutter, 2012). It was based on an earlier model by Tanaka et al., which highlighted a feedback loop involving protein kinase C (PKC), extracellular signal-regulated kinase (ERK) and cytosolic phospholipase A_2_ (cPLA_2_), critical for LTD (Tanaka et al., 2007). The Tanaka model underscored the probabilistic nature of LTD induction, as demonstrated in experimental Ca^2+^ uncaging experiments performed by the same research group. The critical modification of the Antunes model was the inclusion of α-amino-3-hydroxy-5-methyl-4-isoxazole propionic acid (AMPA) receptor trafficking and the prediction that the induction of LTD is all or none. A competing LTD model that combined in a single model the biochemical pathways leading to LTD and the active ion conductances—described in a previously published detailed model (Miyasho et al., 2001)—was described a year earlier (Brown et al., 2011). The Brown model explored the dynamics of phosphatidylinositol-4,5-bisphosphate (PIP_2_) signaling in PCs, which is a molecular pathway element needed for the production of IP_3_ (Brown et al., 2008). However, in contrast to the previously described models (Tanaka et al., 2007; Antunes and De Schutter, 2012), it did not include the positive forward loop in the LTD induction.

Next to models that focused on LTD-relevant molecular pathways, attention was also given to the morphology of the individual dendrite diameters and their impact on the Ca^2+^ diffusion (Anwar et al., 2014). Notably, earlier work from the same research group did not account for the variability of the diameter of the compartment. In the 2014 Anwar et al. paper, the De Schutter laboratory re-examined their earlier work (De Schutter and Bower, 1994b; Anwar et al., 2012), comparing the diffusion errors of 3D and 1D models. They found that the errors from incorrect 3D morphology were higher than the errors caused by the 1D simplification. They also concluded that 3D diffusion is essential for synaptic plasticity and proper modeling of Ca^2+^ signaling pathways.

Multiple synaptic models focused on the mechanisms with which the PCs adapt to external stimuli. Hepburn updated the 2012 synaptic model from De Schutter (Hepburn et al., 2017), to include the Raf kinase inhibitory protein (RKIP) to align with experimental findings (Yamamoto et al., 2012). It also updated the Ca^2+^ dynamics with a fitted rate response to correspond better to existing experimental data. The Hepburn model showed that AMPA receptor (AMPAR) interactions increased robustness of the LTD at the PF-PC synapse, and that LTD probability depended on the amount of PKC. In 2018, the Hepburn model was updated by adding the activation and regulation of the α-calcium/calmodulin-dependent protein kinase II (αCaMKII) (Zamora Chimal and De Schutter, 2018). In accordance with the experimental data (Hansel et al., 2006), LTD in this model was abolished when αCaMKII was not present. Moreover, because ERK, PKC and αCaMKII responded differently to stimuli with varying frequencies, the modeled molecular dynamics of these proteins caused cerebellar LTD to be frequency sensitive.

The TRACE model (Narain et al., 2018), which is based on experimental findings, indicates that interval learning in eyeblink conditioning results in Bayesian-like estimation computed in the cerebellum. The model by Narain has two components: the commonly described LTD, and the less frequently considered long-term potentiation (LTP). The LTD and LTP changes at the GrC-PC synapse changed the PC's SS activity, resulting in a Bayesian Least Squares like estimation through integration of the PC input downstream at the deep cerebellar nuclei (DCN) level. While the Narain model successfully reproduced experimental eyeblink conditioning results, several experimental investigations had shown that the LTD at the PF-PC synapse is not required for cerebellar learning (Schonewille et al., 2011; Johansson et al., 2018). The extent to which LTD is required for cerebellar learning remains the subject of a debate in the cerebellar field.

The experimental data questioning the role of PC-LTD in cerebellar learning led to the development of models which rely on intrinsic plasticity. Majoral and colleagues designed a model in which the Adenylyl cyclase (AC) functioned as a coincidence detector between Ca^2+^ generated from PF input and the G-protein mechanisms triggered by the CF input (Majoral et al., 2020). This coincidence detector functions as a positive feedback loop for low Ca^2+^concentrations, while the higher Ca^2+^ concentrations inhibit the G-protein preventing further activation. The combination of positive and negative feedback loops allows for successful modeling of time intervals. However, the Majoral model simplified the connection between the CF and the dendritic arbor to just the G-protein connection and currently still lacks *in vivo* experimental verification.

The PC has been observed to produce pauses of different durations during eyeblink conditioning, in the absence of MLI inhibition (Johansson et al., 2016). This led Johansson and colleagues to propose intrinsic PC molecular mechanisms as the source of learning. A biochemical pathway that could regulate intrinsic plasticity capable of simulating timing intervals was related to the activation of mGluR7 channels (Mandwal et al., 2021). In this Mandwal's model, response time could be encoded in the rate constants arising from mGluR7 mechanisms. Unique to this model is the description and implementation of the G-protein inward rectifier K^+^ (GIRK) ion channels, whose role in PC-dependent learning also is supported by experimental data (Johansson et al., 2016).

### 2.4 Network models

Purkinje cells' responses vary widely in terms of firing rate modulation, pause duration and morphology. As populations of PCs converge on DCN cells, it becomes relevant to examine collective PC behavior in network models. It is likely that neighboring PCs aligned to PF beams have the highly shared input, which was shown to lead to correlated PC activity *in vivo* (Heck et al., 2007; Cao et al., 2017). Simulations of PC activation over PF beams with the unchanged detailed DB model were used to see if volleys traveling along the beams leads to synchronous active PCs (Jaeger, 2003). About 3 % (~4,500 inputs) of all PFs being active would be sufficient to trigger time-locked PC firing along the PF beam, a fact somewhat aligned with experimental data (Isope and Barbour, 2002). Notably, experimental work presented in the article by Jaeger showed a lack of synchronicity and no narrow cross-correlation peaks between PCs at distances >0.1 mm along the PFs. This discrepancy likely reflects the influence of experimental parameters, notably the PF's feedforward inhibition provided by MLIs (Santamaria et al., 2007). Recent experimental data suggest that even though the PF input might be sparse it can still support very rich stimulus representations (Lanore et al., 2021).

The DB model was also used in a spatial integration study, which analyzed spiking behavior of a single PC receiving different input patterns from PFs and MLIs (Santamaria and Bower, 2005). This study into network dynamics showed that the baseline somatic firing rate of PC is independent from the response to the synaptic inputs. The modulatory effects of the inputs on the SS activity were found to be dominated by dendritic Ca^2+^ and calcium-activated K^+^ currents.

A network model consisting of 50 PCs inhibited by parasagittal projecting MLIs and excited by PFs, yielded PC responses to PF outlasting its input for up to 10 s (Maex and Steuber, 2013). Within this network, PFs delivered transient activity to ‘on-beam' PCs upon a continuous background input over all PFs. Single PCs were based on a trimmed version of the DB model with 196 compartments, enhanced with a modeled axon (Maex and De Schutter, 2007) and an Ih-current (Roth and Häusser, 2001) introduced to all compartments. The trimmed PC model further lacked the resurgent Na^+^ current (Khaliq et al., 2003), and non-inactivating persistent Na^+^ channel. It did however increase the conductance of several channels: (1) the peak conductance of fast Na^+^ channel (NaF) was amplified by 20%; (2) delayed rectifier K^+^ current was doubled; (3) P-type Ca^2+^ and Ca^2+^-dependent K^+^ channels were increased by 40% and 20%, respectively.

The spontaneous firing rate of the 2013 Maex model was set to 71 Hz, but negative current injections would result in very low firing rates. Simulations showed that the PC response only outlasted its stimulus if the PC basic firing rate was in a low frequency range (< 30 Hz). The prolonged excitable states resulted in an increase in membrane voltage compared to simulation runs lacking these excitable states. Further investigation into the observed increased excitability of the PC in this model, revealed that the fast Na^+^ channels (NaF) were responsible for this more depolarised PC state. The level of prolonged excitability was dependent on the PCs basal SS firing rate controlled by PF background activity. Interestingly, the PCs in the 2013 Maex network model showed bistability but only in the sub-millivolt range, which is in stark contrast to the large membrane voltage differences reported by the “classic bistability” PC models (Loewenstein et al., 2005; Fernandez et al., 2007).

Remarkably, other detailed PC models have also been used to study network effects. For example, simulations using the previously described 2012 Forrest model showed that MLI-like GABAergic inputs can switch the PC from a trimodal firing mode into a bimodal firing mode, a finding consistent with experimental observations. Specifically, the GABAergic MLI-like network input was found to be able to block dendritic Ca^2+^ spikes, which would trigger the quiescence.

While the focus of this review is on the PC synapses, other sites of cerebellar plasticity are likely to contribute to motor adaptation. A network model from Clopath et al. (2014) with LTD/LTP plasticity at the PF-PC and plasticity at MF-medial vestibular nuclei (MVN) synapse, simulated vestibular ocular reflex (VOR) adaptation experiments, reliably reproducing experimental data from wildtype mice (Clopath et al., 2014). This model was later used to study learning impairments in six cerebellar mutant mice with impaired motor learning (Badura et al., 2016). The 2014 Clopath model reliably reproduced behavioral deficits as well as PC SS activity abnormalities observed experimentally. Importantly, lack of LTP at the PF-PC broke the Clopath model —where the model does not show any activity— suggesting that it plays an important role in cerebellar-dependent motor learning. This is in line with other experimental evidence showing that LTP is required for procedural learning (Schonewille et al., 2010; Gutierrez-Castellanos et al., 2017).

Electrophysiological measurements of the excitatory (GrC-driven) and inhibitory (MLI- driven) inputs onto the PC were incorporated into a model that explored pre- and postsynaptic plasticity dynamics at the PC synapse (Grangeray-Vilmint et al., 2018). It demonstrated that SS PC firing rate was sensitive to the duration of the GrC burst activity, and that different PCs could either decrease or increase the SS firing upon a similar GrC input, based on the state of the PC synapse. Interestingly, these features were also observed *in vivo* in rhesus macaque PCs during saccadic eye movements (Herzfeld et al., 2015).

In 2019 a spiking cerebellar network model was introduced, where the PCs were based on a modified 2001 Miyasho model, simplified to allow for fast spiking neural network simulations (Luque et al., 2019). This simulated cerebellar network consisted of 100 MFs, 2 CFs, 1,000 GrCs, 20 PCs and 2 MVN cells. It was used to examine how the spike-burst mechanisms can cause LTP blockades during rapid eye movement sleep. This finding was highly relevant to the experimental cerebellar researchers as it offered an explanation for the consolidation of the VOR phase reversal learning, which had been shown to occur overnight (Clopath et al., 2014; Badura et al., 2016). The Luque model also suggested that the pauses following CS bursts in PCs gate the vestibular signals, which accounts for early stages of VOR learning. Altogether, these results predicted that PC SS burst-pause dynamics are instrumental to VOR learning and reversal adaptation. In 2022, a network model for a VOR experiment was performed by Luque and colleagues using Luque's 2019 model. Here, they studied VOR during aging and found that LTP is likely acting as a global homeostatic mechanism that counters age-related vestibular neuroanatomical losses. The 2022 analysis also suggested that VOR is sustained at older ages because the intrinsic plasticity of the PC synapses can operate as a local homeostatic mechanism (Luque et al., 2022).

A different approach to simulating large-scale cerebellar dynamics used modified integrate-and-fire neurons (Fourcaud-Trocmé et al., 2003) to model a network of 50 PCs, 500 MLIs, 500 MFs and 1,000 GrCs (Tang et al., 2021). In this model, each PC received excitatory synaptic input from 100 GrCs and inhibitory input from 8 MLIs. The PCs firing rate and pausing was modulated through a mixture of short term potentiation (STP) on the GrC-PC synaptic connection combined with feedforward inhibition through the MLIs. Extensive simulations showed that the nonlinear characteristics of excitatory STP dynamics significantly modulated PC spiking, mediated by inhibition. In fact, the feedforward inhibition was essential to the PC modulation as the pause response shown in the PC network can only emerge with the interaction of both pathways. This is in line with experimental findings that underscore the essential role of the interaction between excitation and inhibition in the cerebellar cortex (Kim and Augustine, 2021).

In 2023, a network model based on the 2018 Grangeray-Vilmint model was used to investigate a temporal organization of the feedforward inhibitory microcircuit between GrCs, MLIs and PCs (Binda et al., 2023). Using *ex vivo* patch-clamp recordings of PCs and the Grangeray-Vilmint model, it was shown that the encoding of specific MF inputs by PCs can be aided by specific excitatory and inhibitory delays.

The PF-PC synapses undergo both LTP and LTD (though not simultaneously), and these plasticity mechanisms in general counterbalance each other. It has been demonstrated experimentally that different cerebellar zones undergo different opposite directions of plasticity (De Zeeuw, 2021). A recently published olivocerebellar network model incorporated bidirectional plasticity in cerebellar learning by embedding upbound (mainly Z+ PCs) and downbound (mainly Z- PCs) zones that have different plasticity rules (Geminiani et al., 2024). This model showed that plasticity can regulate the cascade of precise spiking patterns spread throughout the CC and DCN.

### 2.5 Perceptron-like models

The earliest neural network that could be trained to distinguish patterns was the perceptron, described in 1958 (Rosenblatt, 1958), building on the earlier work by McCulloch and Pitts (1943). Essentially, a perceptron is a feedforward neural network with threshold units, trained weights and a binary transfer function. The perceptron linearly separates patterns on the basis of supervised learning, and it was taken as the departure point for a tremendously influential Marr-Albus-Ito (MAI) theory of the cerebellum. In short, MAI-based models are based on the premise that the PF-PC synapse undergoes LTP (Eccles et al., 1967) and/or LTD (Albus, 1971) plasticity during learning, which is guided by the CF activity. Within the perceptron framework, the MAI models assume that the modulation of the PF-PC synapse is guided by ‘supervised learning' (CF input), where the weights of each individual synapse are summated and subsequently translated into a PC response. Remarkably, the MAI-type models can also be viewed through the lens of adaptive filter theory (Dean et al., 2010).

When considering the PC as a perceptron, it becomes possible to study the capacity of the cell to encode patterns (Brunel et al., 2004; Clopath et al., 2012; Clopath and Brunel, 2013). An early analysis of the PC as a perceptron model with binary inputs and outputs demonstrated that such a PC can distinguish up to 40,000 patterns, equivalent to 50 KB of storage (Brunel et al., 2004). Furthermore, this model also suggested that in order for the PC to have such a high capacity, many PF-PC synapses would have to be nearly silent (~80% of synapses), in line with experimental data (Isope and Barbour, 2002). Later models improved the estimation of encoding capacity by relaxing the assumption about binary output, allowing firing rate modulation (Clopath et al., 2012; Clopath and Brunel, 2013). Both binary and non-binary models achieved maximum PC storage capacity with up to 80% of the PF-PC synapses being silent. The 2012 model of Clopath also quantified the capacity for pattern separation afforded by bistable firing. The CF signal not only affected supervised learning triggering the weight changes, but could also switch the PCs from up to down states. Bistability enhanced the PC storage capacity and predicted that for maximal storage capacity the bistable range must increase when correlation between input patterns decreases. Clopath and Brunel updated the perceptron model one more time in their 2013 paper to incorporate more physiologically relevant input and output patterns represented by frequency trains (analog signals) rather than binary on/off signals. Interestingly, the learning capacity of the 2013 model was highest when the input variance was the largest. This type of variance can be found experimentally in GrC firing behavior—low mean firing rate with high frequency bursts (Chadderton et al., 2004; Jörntell and Hansel, 2006; Rancz et al., 2007).

It is also possible to study PC encoding capacity in highly detailed compartmental PC models (Steuber and Schutter, 2001; Steuber et al., 2007). The Steuber models relied on the standard MAI-based LTD induction principle, where the PF input pattern was ‘trained' under the CF supervision. These studies were based on the morphologically and biophysically detailed PC model discussed previously (De Schutter and Bower, 1994a,b). Continuous excitatory background input to the DB model resulted in biologically realistic PC firing rates of 48 Hz. Novel input patterns were found to trigger higher responses than learned input patterns, and active membrane conductances in the dendrites were found to increase the discrimination between novel and learned input patterns (Steuber and Schutter, 2001). Whereas, both novel and learned stimuli triggered an initial SS burst, the pause length following the burst was predictive of the type of the stimulus. Analysis of PC responses in the 2007 Steuber model found that a hyperpolarizing Ca^2+^ triggered K^+^ efflux underlied the SS pause. Thus, LTD would encode patterns by shortening pauses, a prediction that has not yet been experimentally verified. Pattern recognition based on pause length showed that PF patterns between 750 to 8,000 active PFs allowed reliable discrimination. Patterns with PF activity levels outside of this range failed to reliably differentiate between input based on the pause length.

There are also other encoding schemes available to the PC beyond the duration of pause modulation. In 2009 Walter analyzed firing frequency as coding strategy of the PC to differentiate learned from novel input patterns (Walter and Khodakhah, 2009). The perceptron-like network model from which the coding capacity was deduced was based on the 2007 Steuber model. The Walter model simulated a PC innervated by 150,000 independent GrC synapses. A number of 650 synapses in a specific pattern was calculated to be able to trigger the maximal firing rate increase in PCs. In other words, increasing input from 0 to 650 PF's would take a PC from 50 to 250 Hz, revealing the large and linear dynamic range of the PC response. The authors convincingly argued that the firing rate frequency output gave the PC with a better coding capacity than pause duration. Moreover, asynchronous activation of synaptic input patterns over 10–25 ms windows showed a significantly better coding capacity if the PC used the firing rate coding strategy as compared to pause induction. A firing rate strategy also showed increased performance if a base level firing was introduced, which was not present in the 2007 Steuber model.

## 3 Discussion

**Figure 3:**
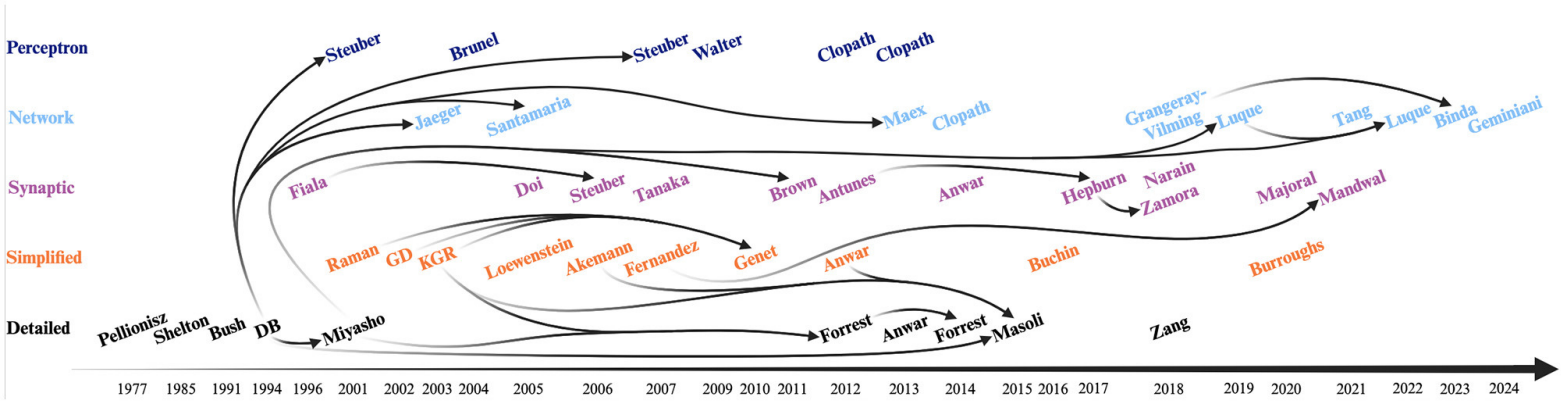
Timeline of selected Purkinje cell (PC) models. Each color represents a different category: detailed (black), simplified (orange), synaptic (purple), network (light blue) and perceptron-like (dark blue) models. Initial models were all detailed models. The Zang et al. (2018) model was designed as a modern update to the original 1994 De Schutter and Bower (DB) model. The DB model was the basis not only for the first network (Jaeger, 2003; Santamaria and Bower, 2005; Maex and Steuber, 2013) and perceptron-like (Steuber and Schutter, 2001; Steuber et al., 2007) models, but as well directly or indirectly for the majority of the next detailed models—except for the Anwar model. The Miyasho et al. (2001) model (based on DB model) was the basis for further synaptic (Brown et al., 2011; Luque et al., 2019, 2022) network models. The appearance of simplified models such as the Raman and Bean (2001), Genet and Delord (2002) (GD), Khaliq et al. (2003) (KGR), and Fernandez et al. (2007) models allowed for further advancement for detailed models such as the Forrest et al. (2012) and Masoli et al. (2015) models, for other simplified models such as the Genet et al. (2010) model, and for synaptic models such as the Mandwal et al. (2021) model. The Fiala et al. (1996) and Antunes and De Schutter (2012) synaptic models were used as basis for other synaptic models: the Steuber et al. (2006) and Hepburn et al. (2017) (in turn used for the Zamora Chimal and De Schutter, 2018 model), respectively. Finally, the Binda et al. (2023) network model was based on the Grangeray-Vilmint et al. (2018) model of the same type. Figure generated with BioRender.

Models of the PC capture much of the complex phenomenology of this cerebellar cell, though not all at the same time. Modeling is the art of selecting what and how to explain. In the context of the PC, as with many other biological systems, there are many levels at which a given phenomenon can be elucidated. Sometimes a simple model is the most effective method to understand the mechanism underlying a behavior, but in other cases the full phenomenology can be captured accurately only with added detail. There is an undeniable tension between the goals of simple vs accurate (detailed) models, but as we see in this review, their explanatory powers are often complementary. In a sense, it is this model “ensemble” (Figure 3) that describes intrinsic PC activity as well as its function in the context of a network.

A common criticism of biophysically plausible computational modeling is that much of the modeling work is geared toward reproducing behavior known from experiment. It is also often suggested that biophysically plausible modeling scarcely produces experimentally testable hypotheses. This criticism is sufficiently prevalent that merits discussion. What is gained by capturing complex behavior via parameters that represent specific conductances is a quantitative understanding of the spectrum of neuronal dynamics, which cannot be fully captured in one experiment. The goal of modeling is to generalize to cover a whole class of neurons, and their potential transitions across modes of activity. All models are incomplete, in the sense that not all relevant mechanisms that explain cell activity are incorporated in a single model (i.e. metabolism, transcription, regulation), and many important factors are, by necessity, excluded. Nevertheless, modeling work has also led to a profusion of experimental hypotheses. All the same, one can argue that one of the principal roles of biophysically plausible modeling is to produce *mechanistic* and *computational* understanding. Indeed, we argue that many models discussed in this review offer explanations for specific biologically observed phenomena.

In terms of the PCs computational role, modeling studies often converge on three main coding mechanisms: (1) a rather linear firing rate response to current input, (2) a complex set of intracellular Ca^2+^ dynamics that induce plasticity, and (3) tuneable SS pause durations. The fact that a relatively small number of active PF synapses can drive the PC response from minimum to maximum means that PCs exhibit a relatively narrow dynamical range, in the sense of signal analysis. It is still unknown whether silent synapses are a transient condition, or if they can be rapidly enlisted in short timeframes (Barbour, 1993). In any case, the small proportion of active PF synapses is still a befuddling fact of PC physiology and function.

One important point about levels of analysis is that the computational properties of the cell are not equal to its dynamics and mechanisms. Regardless of the functional roles of a PC in a network, their models may not need to capture the entire repertoire of biological detail. It is conceivable that adding specific detail may actually be detrimental to its computational role in the circuit, even if it reproduces experiment with exquisite precision. On the one hand, exploring the parameter space in which a model can no longer produce meaningful data is important to understanding its limitations. On the other hand, it is important to be aware of the bias contained in the statement that “because something exists in biology, it must play a functional role”, what Gould and Lewontin called “adaptationism” (S. J. Gould and Lewontin, 1979). Nevertheless, the value of an accurate description of a neuron cannot be understated, as it gives insight into healthy cellular dynamics and gives us confidence that the mechanisms we observe contribute to the overall electrophysiological behavior of the cell.

There are several potential caveats to the PC models presented in this review. First, most PC models are based on electrophysiological data obtained from *ex-vivo* slice recordings or *in-vivo* recordings under anesthesia. As discussed briefly in section 2.2.1 “Bistability”, anesthesia affects neuronal channel conductances, most often leading to reduced firing. The most commonly used method is isoflurane inhalation anesthesia, which at clinically relevant concentrations, leads to membrane hyperpolarization via its effects on the Na^+^ voltage-dependent channels (Qiu et al., 2023). Notably, isoflurane replaced an older method of urethane inhalation anesthesia, which acted on K^+^ voltage-dependent channels, and was shown to lead to cycling between up and down states (Yagishita et al., 2020). The second most commonly used sedation method is a ketamine/xylazine cocktail injected intraperitoneally, which is a mixture on an NMDA receptor antagonist (ketamine) and an α2-adrenergic receptor agonist (xylazine). On the one hand, all these methods affect neuronal electrophysiological properties, which in turn might lead to improper inputs to the PC models. On the other hand the very same models could be used to study different classes of the anesthetics and their effects on cerebellar function. Nevertheless, anesthesia is a factor that can introduce many unforeseen variables. Therefore newer models, particularly the ones that describe network activity during learning, are largely based on data from *in-vivo* awake recordings from behaving animals. Second, our review focuses primarily on models of the PC itself, and thereby excludes most of the circuit resonances feeding back into the CC such as: the Golgi cell oscillations, PC to PC inhibition, DCN feedback to GrCs, the IO dynamics and the feedforward inhibition of MLIs. Particularly, the significant impact of the MLI inputs on PC activity and cerebellar learning has been extensively researched in the last decade (Badura et al., 2013; Brown et al., 2019; Ma et al., 2020), but has not yet been investigated in depth in the modeling work, with the notable exception of network models. Overall, complex network dynamics make the interpretation of the experimental data, restricted to PC recordings, difficult. However, a good grasp of the mechanisms underlying activity of the PC in isolation, is instrumental to disentangle complex cerebellar network responses. Third, even when models include large numbers of conductances and other dynamics, publications often focus on a few mechanisms at a time. This is partly due to the difficulty of visualizing high dimensional parameter spaces, and partly due to the fact that adding new mechanisms exponentially expands parameter space, which is computationally costly. This is one of the reasons why there is no ‘final model' of the PC, which includes ‘everything'. Computational scientists have a preference to study only the mechanisms that are required for a given behavior of interest. Finally, with the rise of the new era of big omics, cerebellar sequencing data has revealed that there might in fact be several categories of PCs with different characteristics and properties (Kozareva et al., 2021; Apsley and Becker, 2022). However, at present these new categories are not yet linked to excitability differences, or other functional or encoding correlates. One fundamental open question for computational modeling of the PC is the extent to which excitability differences found in distinct Zebrin zones can be linked to specific model parameters. It seems promising that highly constrained biophysical models can help us express the different categories of cells as they relate to parameter ranges. We foresee a future in which complex neuron models will be able to help us link results from the genomics and proteomics studies with neuronal dynamics.

As computational neuroscience moves forward, the key issue for upcoming research is the development of accurate models that give us insight into necessary homeostatic mechanisms of complex neurons such as the PC. Tuning accurate models reproducing specific behaviors at the level of cells and organisms, with plausible models of cellular dynamics, gives researchers a handle on how to interpret neuronal activity in health and disease. Future models must tackle the question of how to extrapolate from single detailed models to entire populations close to the biological constraints, particularly across different brain regions. As neurons need to “change to stay the same” (Abadia et al., 2021), the inclusion of homeostatic mechanisms in the current models is a promising research direction, so that we better understand how a cell changes within functional constraints.

Finally, the question should be addressed of whether abstract PC models are sufficient to represent motor learning. Though robotics applications have been using the Perceptron analogy for a long time (Vijayan et al., 2017), there is no “full spectrum” theory of motor learning on the basis of PCs as Perceptrons. It seems that the ways in which PCs are used in biological brains will still be providing ample insight to develop computational models and potential future AI applications to represent biological learning.

## Author contributions

EF: Writing – original draft, Writing – review & editing. AK: Writing – original draft, Writing – review & editing. PW: Writing – original draft, Writing – review & editing. CD: Funding acquisition, Writing – original draft, Writing – review & editing. AB: Funding acquisition, Project administration, Resources, Supervision, Writing – original draft, Writing – review & editing. MN: Conceptualization, Funding acquisition, Investigation, Methodology, Project administration, Resources, Supervision, Writing – original draft, Writing – review & editing.
